# Investigation on the Effect of Annealing Temperature on the Side Ohmic Contact Characteristics for Double Channel GaN/AlGaN Epitaxial Layer

**DOI:** 10.3390/mi13050791

**Published:** 2022-05-19

**Authors:** Qingzhi Meng, Qijing Lin, Weixuan Jing, Na Zhao, Ping Yang, Dejiang Lu

**Affiliations:** 1 State Key Laboratory for Manufacturing Systems Engineering, Xi’an Jiaotong University, Xi’an 710049, China; qzmeng2016@foxmail.com (Q.M.); wxjing@mail.xjtu.edu.cn (W.J.); zn2015@stu.xjtu.edu.cn (N.Z.); ipe@xjtu.edu.cn (P.Y.); djlu@xjtu.edu.cn (D.L.); 2 Collaborative Innovation Center of High-End Manufacturing Equipment, Xi’an Jiaotong University, Xi’an 710054, China; 3 School of Mechanical and Manufacturing Engineering, Xiamen Institute of Technology, Xiamen 361021, China; 4 Chongqing Key Laboratory of Micro-Nano Systems and Intelligent Sensing, Chongqing Academician Workstation, Chongqing 2011 Collaborative Innovation Center of Micro/Nano Sensing and Intelligent Ecological Internet of Things, Chongqing Technology and Business University, Chongqing 400067, China

**Keywords:** side ohmic contact, transmission line model, specific contact resistance, annealing temperatures

## Abstract

A side ohmic contact mode for the double channel GaN/AlGaN epitaxial layer is proposed in this paper. Rectangle transmission line model (TLM) electrodes are prepared, and the specific contact resistance is tested at the annealing temperatures from 700 °C to 850 °C. The results show that the minimum specific contact resistance is 2.58 × 10^−7^ Ω·cm^2^ at the annealing temperature of 750 °C, which is three to four times lower than the surface contact mode. Scanning electron microscope (SEM), energy dispersive spectrometer (EDS), and atomic force microscope (AFM) were carried out for the analysis of the morphology, element composition, and the height fluctuation at the contact edge. With the increase in the annealing temperature, the specific contact resistance decreases due to the alloying of electrodes and the raised number of N vacancies. However, when the annealing temperature exceeds 800 °C, the state of the stress in the electrode films transforms from compressive stress to tensile stress. Besides, the volume expansion of metal electrode film and the increase in the roughness at the contact edge leads to the degradation of the side ohmic contact characteristics.

## 1. Introduction

With the development of microwave communication technology, higher requirements are put forward for the frequency and power characteristics of microelectronic devices. GaN-based devices, such as GaN/AlGaN high electron mobility transistors (HEMT) and GaN diodes, have been widely used as power devices because of their high breakdown voltage [1,2,3] and operation frequency [4,5,6]. Although GaN devices have many advantages, they are still suffering from some problems such as short channel effect [7,8,9] and current collapse effect [10,11,12]. To solve these problems, some researchers proposed double channel GaN HEMTs [13,14,15,16,17,18], which contain two GaN/AlGaN heterojunction conductive channels. For devices with such structures, the current collapse effect is significantly weakened, as the lower barrier layer is far from the surface of the epitaxial layer. Besides, since double channel GaN HEMTs have a stronger confinement ability of carriers, they have higher breakdown voltage, and the carrier transport capacity is almost twice that of single channel devices [16,17].

However, the introduction of an additional channel will simultaneously involve some defects. For GaN HEMTs, the ohmic contact between a source/drain electrode and epitaxial layer is very important, as it will greatly affect the output characteristics of the device. When a positive bias is applied between source and drain, electrons firstly tunneling from the source to the conductive channel are then driven to the drain terminal. In this process, the tunneling probability, depending on the height of the barrier, plays an important role. Especially for double channel GaN HEMTs, the lower channel and source electrode are separated by two AlGaN barrier layers and a GaN channel layer, which increases the barrier height and decreases the tunneling probability of electrons. To overcome this problem, researchers have put forward the side ohmic contact mode. Metal electrodes are deposited on the side of the epitaxial layer to put electrodes directly in contact with the 2DEG conductive channel, which greatly reduces the contact resistance and improves the tunneling probability of electrons. This contact mode has already been used in single channel GaN HEMTs [19,20,21]. Luan et al. investigated the polarization Coulomb field (PCF) scattering in AlGaN/AlN/GaN HEMT [19] and InGaN/AlN/GaN HEMT [20] with side ohmic contact and found that the PCF scattering was greatly weakened in side ohmic contact mode compared with normal surface contact mode, and the two-dimensional electron gas (2DEG) density was also enhanced. Wang et al. [21] studied the effect of the etching process on the side ohmic contact performance and revealed that the carrier transport is more efficient in side ohmic contact than the surface contact mode. It is believed this contact mode could also be utilized in double channel GaN HEMTs to improve the tunneling probability, especially in the lower channel, to decrease the specific contact resistivity. Furthermore, if the side ohmic contact mode can improve the output characteristics of GaN HEMTs in the RF circuit, the power amplification performance would be promoted [22]. Some photodetectors [23,24,25] or sensors [26,27,28] based on GaN HEMTs also have high requirements for their output characteristics. For instance, the background noise voltage of GaN HEMT terahertz detector is related to the resistance of the source to drain conductive channel, and the outstanding ohmic contact characteristics can obtain smaller noise equivalent power [23]. For a GaN HEMT hydrogen sensor, the variation of drain current *I_ds_* depends on the concentration of hydrogen [28], so the better the ohmic contact of drain and source electrodes is, the higher the sensitivity of the sensor will be. Therefore, it is necessary to study the side ohmic contact characteristics between the double channel GaN/AlGaN epitaxial layer and metal electrodes to improve the output performance of double channel GaN HEMTs. For the conventional surface contact mode, the annealing condition for the ohmic contact between a metal electrode and GaN substrate is, basically, explicitly to rapidly anneal for 30 s at the temperature of 850 °C. However, the mechanism of the side contact mode is different from the surface contact mode, the annealing temperature for the formation of ohmic contact is different. Besides, as the depth of the groove structure of side ohmic contact mode for the double channel GaN/AlGaN epitaxial layer is deeper than single channel, it may cause the lift-off of the electrode from the side of the epitaxial layer and the degradation of the ohmic contact, which is a challenge for the preparation of the side ohmic contact of the double channel GaN/AlGaN epitaxial layer and/or double channel GaN HEMT. The motivation of this paper is to explore the optimal annealing temperature of side ohmic contact mode for the double channel GaN epitaxial layer to obtain the lowest specific contact resistance between a metal electrode and the epitaxial layer.

In this paper, side ohmic contact electrodes were prepared on the double channel GaN/AlGaN epitaxial layer by magnetron sputtering technology, and they were annealed at temperatures from 700 °C to 850 °C. The specific contact resistance was tested by the transmission line model (TLM). The analysis of metal electrode films was carried out by a scanning electron microscope (SEM), an energy dispersive spectrometer (EDS), and an atomic force microscope (AFM), and the side ohmic contact performance at different annealing temperature was investigated.

## 2. The Measurement Method of the Specific Contact Resistance

**Figure 1:**
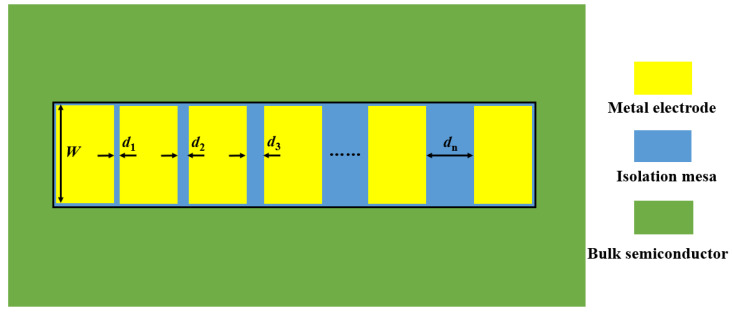
The schematic diagram of the rectangular electrode TLM structure.

TLM is the most commonly used method to measure the specific contact resistance. The TLM method can be divided into circular electrode TLM and rectangular electrode TLM method. For circular electrode TLM method, the metal electrode can be directly deposited onto the bulk material without the formation of the mesa, but the calculation of the specific contact resistance is complicated, while, for rectangular electrode TLM method, it is a simple and accurate way to calculate the specific contact resistance, even though an isolated mesa needs to be prepared. The schematic diagram of the rectangular electrode TLM structure is shown in Figure 1. To make electrons transfer along the horizontal direction, a rectangular isolation mesa is firstly prepared on the bulk semiconductor, and then, several rectangular metal electrodes with arithmetic sequence distance are deposited on the isolation mesa.

**Figure 2:**
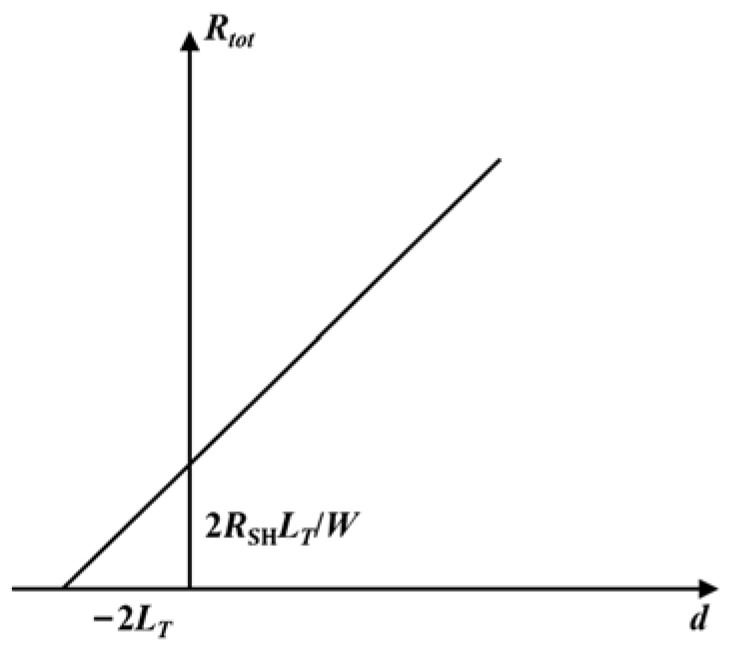
The curve of *R_tot_* versus *d*. The slope of the curve is *R_SH_*/*W*, and the intercept is 2*R_SH_L_T_*/*W*.

According to the TLM theory, the total resistance between each adjacent electrode in Figure 1 is given as:

(1) $$R_{tot}=2R_{c}+R_{SH}\frac{d}{W}$$

where *R_tot_* is the total resistance, *R_SH_* is the sheet resistance of the semiconductor, *L_T_* = $\sqrt{\rho_{c}/R_{SH}}$ is the transmission length, *W* is the width of electrodes, and *d* is the distance between adjacent electrodes. By measuring the I-V characteristics of electrodes with different distances, the curve of *R_tot_* versus *d* can be plotted, as with Figure 2, where the slope of the curve is *R_SH_*/*W*, and the intercept is 2*R_SH_L_T_*/*W*. The *R_SH_* and the *L_T_* can be obtained, and the specific contact resistance $\rho_{c}$ is calculated as $\rho_{c}=L_{T}{}^{2}R_{SH}$.

## 3. Experimental Procedure

### 3.1. The Growth of Double Channel GaN/AlGaN Epitaxial Layer

**Figure 3:**
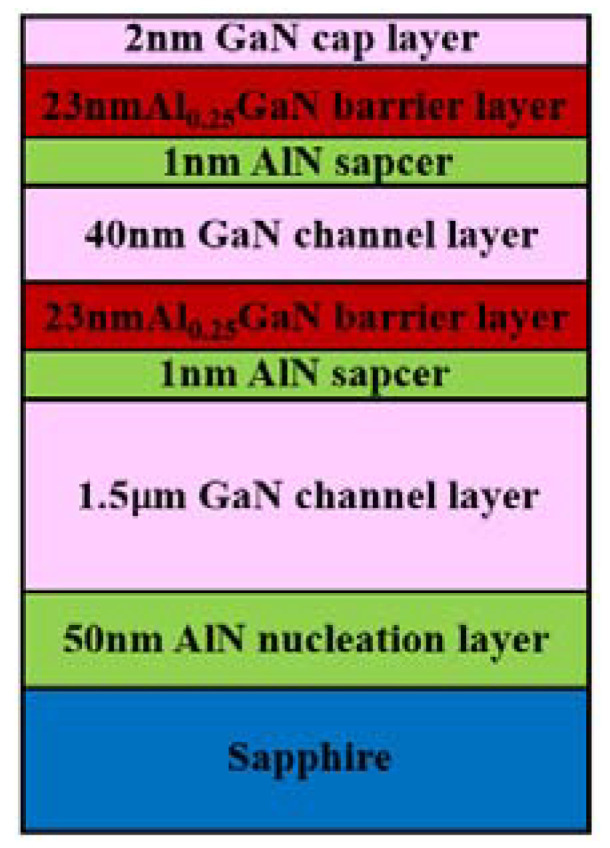
Diagram of the double channel epitaxial layer.

Figure 3 shows the diagram of double channel GaN/AlGaN structures. GaN/AlGaN epitaxial layers were grown by metal organic chemical vapor deposition (MOCVD) technology. Triethylgallium (TEGa), trimethyl aluminium (TMAl), and ammonia (NH_3_) were used as the sources of Ga, Al, and N, respectively. High-purity hydrogen (H_2_) was used as the carrier gas. A 50 nm AlN nucleation layer was firstly deposited on c-axis plane (0001) sapphire substrates, and then, a 1.5μm GaN channel layer was grown, followed by a 1 nm AlN spacer and 23 nm Al_0.25_GaN barrier layer to form the lower channel. Afterwards, the upper channel was formed by the growth of 40 nm GaN channel layer, 1 nm AlN spacer, and 23 nm Al_0.25_GaN barrier layer. Finally, a 2 nm GaN cap layer is grown on the top of the epitaxial layer surface. Detailed results of the material parameters and electrical parameters for the epitaxial layer are in our previous work [29]. The average electron mobility of the epitaxial layers is 1815 cm^2^/V·s^−1^, and the sheet density of carriers is 8.247 × 10^12^/cm^2^.

### 3.2. The Prepartion of TLM Electrodes

**Figure 4:**
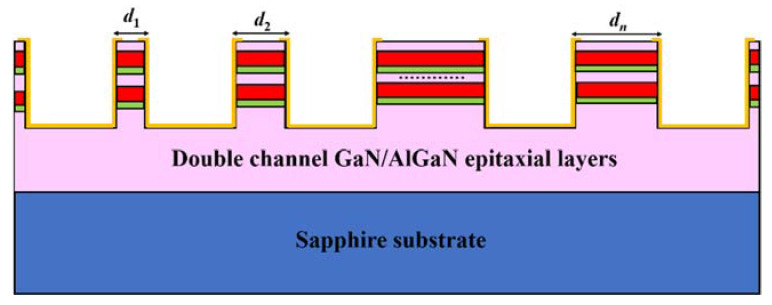
The cross section of the side ohmic contact mode. The pink region refers to the GaN layer, the red region refers to the AlGaN layer, and the green region refers to the AlN layer.

The cross section of the side ohmic contact mode is shown in Figure 4. Compared with the conventional surface contact mode, electrodes are deposited on the side of the conductive channel, instead of the surface of the epitaxial layer, to reduce the contact barrier between the electrode and the channel. For the I-V characteristics measurement of TLM electrodes, the distance between each electrode is in an arithmetic sequence.

**Figure 5:**
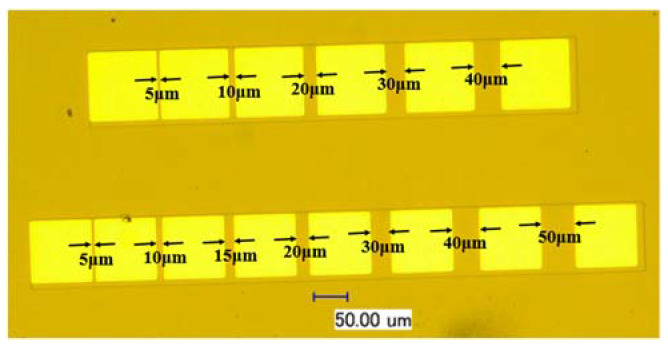
The top view of the prepared TLM electrodes samples.

The isolation mesa and the groove structures were etched by inductive coupled plasma (ICP) (Corial, 210 L, Bernin, France) technology. BCl_3_, Cl_2_, and Ar were used as the etching gases. Main parameters used in the etching process are shown in Table 1. The Ti/Al/Ni/Au (20 nm/120 nm/40 nm/50 nm) metal film electrodes were deposited on the side of the channel using magnetron sputtering (Denton, Dsicovery635, Beijing, China) technology. Then, the samples were annealed in the rapid annealing furnace (UNTIMP, RTP-100, Pfaffenhofen, Bavaria, Germany) with N_2_ atmosphere for 30 s at the temperature of 700 °C, 750 °C, 800 °C, and 850 °C, respectively. Finally, the I-V characteristics were tested by a semiconductor device analyzer (KEYSIGHT, B1500A, Santa Rosa, CA, USA). The prepared TLM electrode samples are shown in Figure 5.

## 4. Results and Discussion

**Figure 6:**
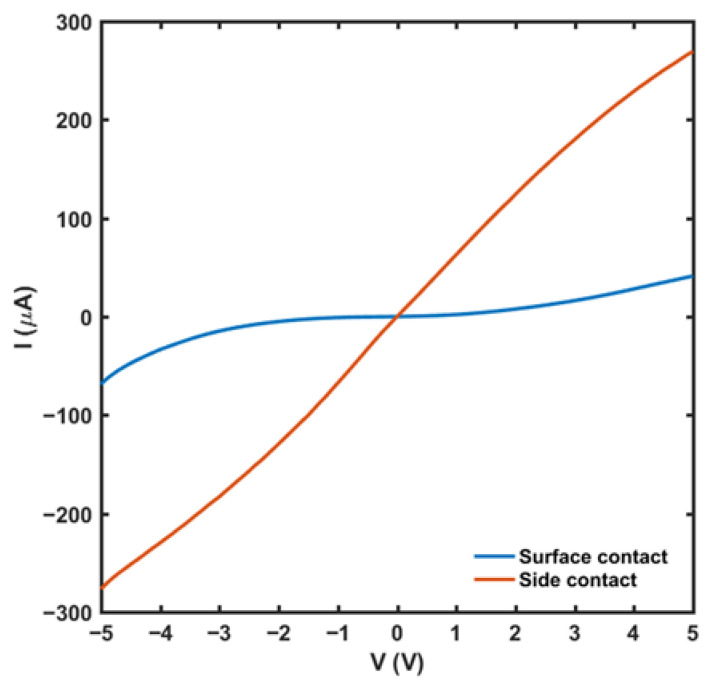
The I-V characteristics of the surface contact and side contact mode with the electrode distance of 40 μm when not annealed.

Firstly, the I-V characteristics of the unannealed samples were tested. Figure 6 shows the I-V characteristic curves of the surface ohmic contact mode and side ohmic contact mode. It can be seen that, for the surface contact mode, the contact mode between the metal electrodes and epitaxial layer is Schottky contact before annealing, and the ohmic contact has not been formed yet, while for the side contact mode, the I-V curves have already shown the features of ohmic contact. This is because, in the surface contact mode, there is no alloying between the electrodes and the surface of the epitaxial layer when not annealed, and the barrier between electrodes and the channel is too high for electrons to jump over. However, metal depositing on the side of the epitaxial layer can make the electrode directly contact the conductive channel, which significantly reduces the contact barrier. After annealing at a suitable temperature, the contact resistance between the electrode and the side of the epitaxial layer will be further reduced, leading to the formation of a good ohmic contact.

**Figure 7:**
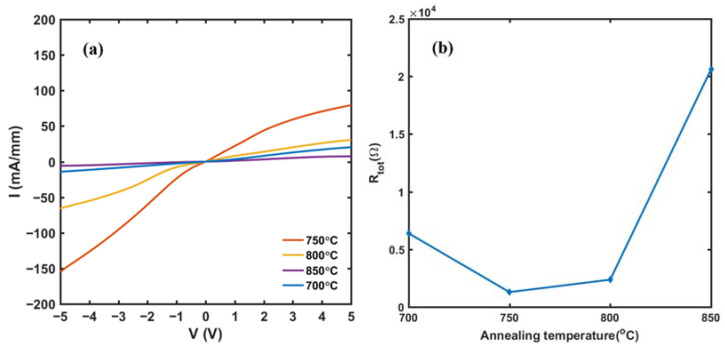
(**a**) I-V characteristics of side ohmic contact TLM electrodes, with a distance of 40 μm at different annealing temperatures. (**b**) The total resistance of side ohmic contact TLM electrodes with the change of annealing temperature.

**Figure 8:**
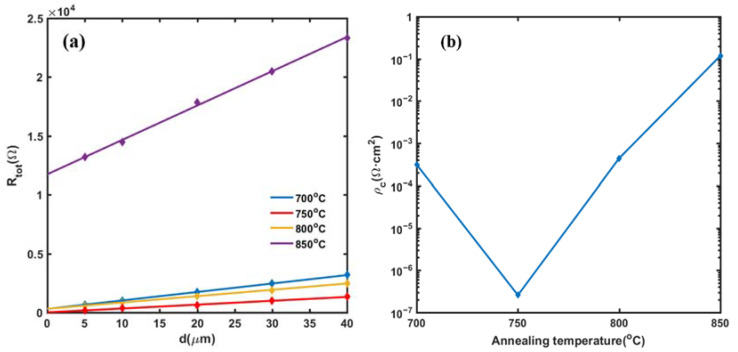
(**a**) The total resistance with the change of the distance between side ohmic contact TLM electrodes. (**b**) The specific contact resistance with the change of annealing temperature.

The I-V characteristics curves of the side ohmic contact samples at the annealing temperature, from 700 °C to 850 °C, are shown in Figure 7a. The current rises linearly with the increase in voltage, which proves that the side contact mode can form the ohmic contact between metal electrodes and the conductive channel after rapid annealing. Figure 7b shows the total resistance *R_tot_* with the change of temperature from 700 °C to 850 °C. The *R_tot_* is minimum at the annealing temperature of 750 °C, after which the *R_tot_* begins to increase with the annealing temperature. From these results, it can be preliminarily determined that 750 °C is the optimal annealing temperature. After measuring the I-V characteristics of side ohmic contact electrodes with different spacing, the *R_tot_*-*d* curves were plotted in Figure 8a, from which the specific contact resistance $\rho_{c}$ can be extracted by Equation (1). Figure 8b shows the calculated $\rho_{c}$ at a different annealing temperature, where the minimum $\rho_{c}$ is as low as 2.58 × 10^−7^ Ω·cm^2^ at the annealing temperature of 750 °C. This value is three to four times lower than the specific contact resistance of 1 × 10^−6^ Ω·cm^2^ in our previous prepared GaN HEMT device [23], as well as most of the reported surface contact mode [30,31,32,33]. The results show that, for a double channel GaN/AlGaN epitaxial layer, the side ohmic contact mode can obtain smaller specific contact resistance than surface contact mode (e.g., 3.29 × 10^6^ Ω·cm^2^ in [30], 2.54 × 10^−6^ Ω·cm^2^ in [31], 1.35 × 10^−6^ Ω·cm^2^ in [32], and 3.7 × 10^−6^ Ω·cm^2^ in [33]).

**Figure 9:**
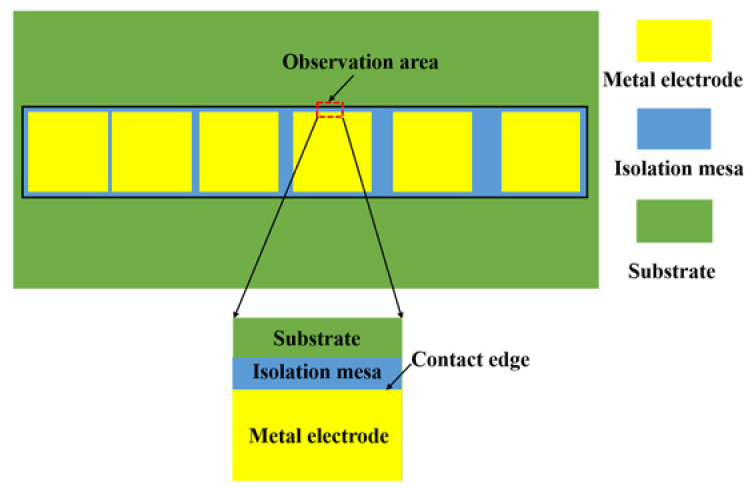
Schematic diagram of electrode edge area scanned by SEM.

**Figure 10:**
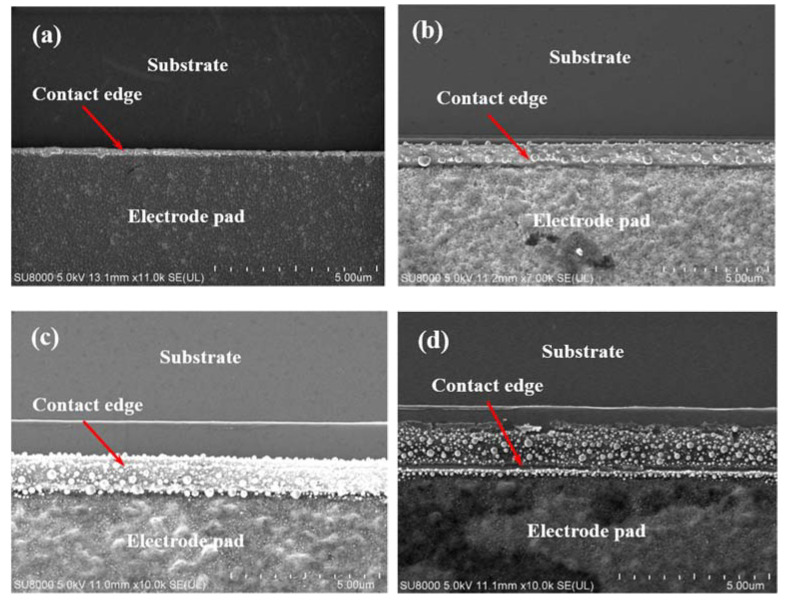
The SEM images of TLM electrode morphology at the annealing temperature of (**a**) 700 °C, (**b**) 750 °C, (**c**) 800 °C, and (**d**) 850 °C.

In order to further investigate the ohmic contact effect of TLM electrodes at different annealing temperatures, the morphology of TLM electrodes was observed. The ohmic contact is formed on the side of the epitaxial layer, so we mainly focused on the boundary of electrode and mesa, as shown in the marked region of Figure 9. Figure 10 shows the planar SEM images of the electrode edge (marked region in Figure 9). When the annealing temperature is 700 °C, the metal electrode well contacts the side on the channel, and the edge morphology is flat and smooth. When the annealing temperature is 750 °C, some small grains are formed on the contact edge. These grains are the products of the reaction of metal layers at high temperatures, which indicates the formation of the alloying. When the annealing temperature is 800 °C, the number of grains becomes more. When the annealing temperature reaches 850 °C, there cracks appear at the contact edge and even cause the lift-off of the electrode. From Figure 10a–d, it can be noted that the stress in the Ti/Al/Ni/Au films changes with the increasing of the annealing temperature, which leads to the variation in the ohmic contact characteristics. Films initially sputtered at room temperature show a state of compressive stress due to the shot peening action of the bombarding ions and neutrals [34]. With the increase of the annealing temperature, Ti reacts with GaN/AlGaN and generates TiN to create N vacancies and form the ohmic contact. However, the Al also reacts with Ni and produces NiAl_x_, which makes the stress move into the tensile state and weaken the film adhesion. This may be the reason why the ohmic contact characteristics first improve and then degenerate with the increase in annealing temperature. If the annealing temperature continues to rise, microcracks, together with the volume expansion of the interface, will make the electrode fall off the side of the epitaxial layer (Figure 10d).

**Figure 11:**
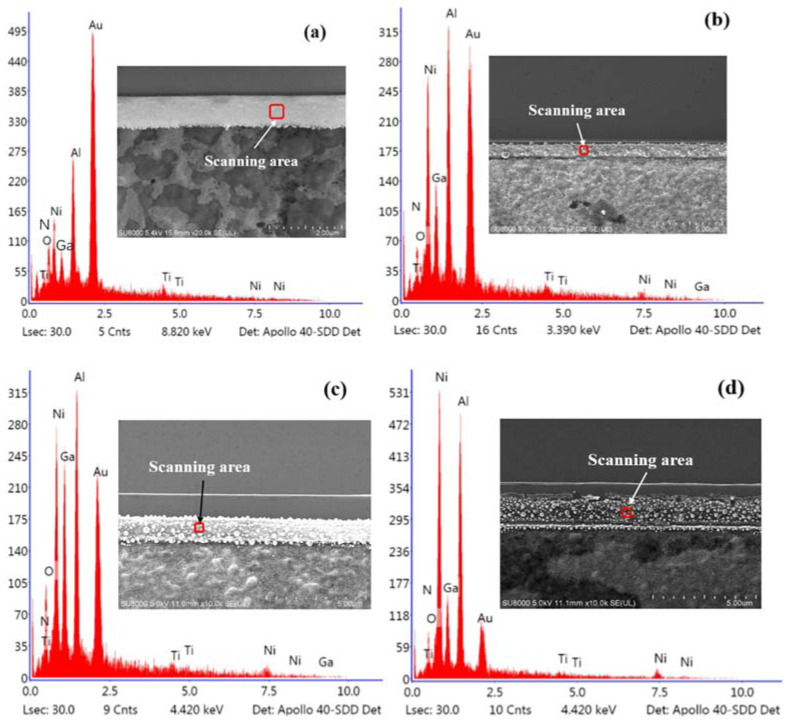
The EDS spectra of TLM electrodes at the annealing temperature of (**a**) 700 °C, (**b**) 750 °C, (**c**) 800 °C, and (**d**) 850 °C.

To evaluate the composition of the annealed metal films, the EDS spectra was carried out simultaneously with the observation of the SEM. The side contact region that we mainly focused on was taken for the EDS spectra scanning (in the red circle area), and the results were shown in Figure 11. At the annealing temperature of 700 °C (Figure 11a), the Au component is dominant, as the Au layer is on the surface of metal layers, and no cracks or diffusion of metal layers appear. The Ga and N element is small but distinguishable because the AlGaN barrier is beyond the metal layers, and the annealing temperature is not high enough to make N element diffuse into the metal layer and generate N vacancies, so the specific contact resistance is large. When the annealing temperature reaches 750 °C (Figure 11b), the content of N is 20.76%, which is much higher than that of the 700 °C annealing temperature. The N element includes the compounds in the GaN/AlGaN epitaxial layer and TiN formed by high temperature annealing. The increase in N content indicates that N element diffuses into the upper metal layer and creates TiN, and the ohmic contacts are well formatted. When the annealing temperature reaches 800 °C (Figure 11c), the content of N increases to 30.74%, but the content of Au decreases sharply from 21.49% to 8.1%. This indicates the decrease in adhesion and the expansion of volume causes the lift-off of the Au film and the degradation of ohmic contact characteristics. When the annealing temperature is 850 °C (Figure 11d), the content of Au decreases to 2.82%, and the specific contact resistance increases significantly.

**Figure 12:**
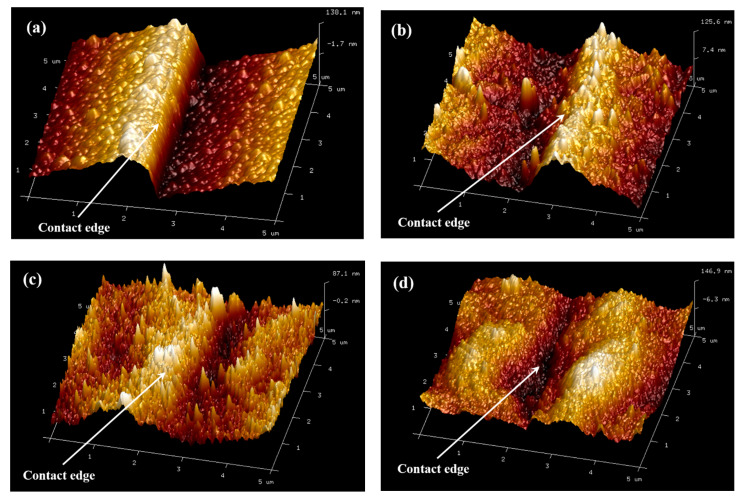
The morphology of the contact edge between the metal electrode and the side of the epitaxial layer, measured by AFM with the scanning area of 5 × 5 μm, after annealing at the temperature of (**a**) 700 °C, (**b**) 750 °C, (**c**) 800 °C, and (**d**) 850 °C.

**Figure 13:**
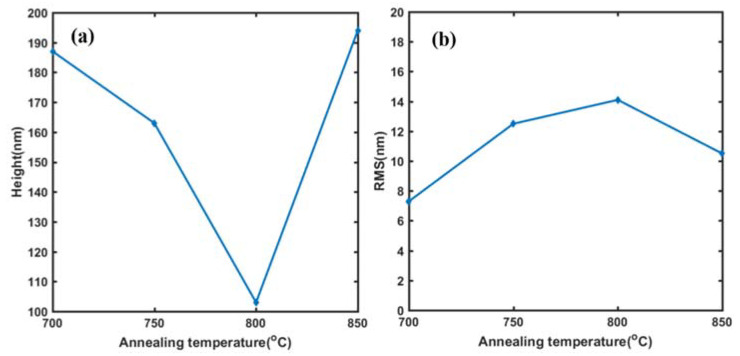
The step height (**a**) and the RMS (**b**) at the contact edge of the electrode and the epitaxial layer at the annealing temperature from 700 °C to 850 °C.

To further study the morphology variation of the contact between the metal electrode and the side of the epitaxial layer after high temperature annealing, the AFM was carried out for the quantitative analysis of the height fluctuation at the contact edge. From the three-dimensional image of the contact edge morphology in Figure 12a,b, it can basically be seen that, with the increasing of the annealing temperature, the morphology of the contact edge turns to be more and more rough, and cracks even appear at the annealing temperature of 850 °C. Figure 13 plots the variation of the step height (Figure 13a) and the root mean square roughness (RMS) (Figure 13b) of the contact edge with annealing temperature. At the annealing temperature of 700 °C, the step between the electrode and the mesa is clearly distinguishable, and the sectional measurement results show that the height of the stage is 187 nm, which is a little lower than the total thickness of the deposited metal layers. At the annealing temperature range of 700–800 °C, the step height decreases with the annealing temperature, while the RMS increases with the annealing temperature. This is possibly due to the change of the stress state and the volume expansion of metal films at high temperatures and leads to the reduction in the step height. On the other hand, with the increasing of the annealing temperature, the alloying of metals becomes more and more obvious and creates grains on the side, which makes the roughness increase. When the annealing temperature reaches 850 °C, metal layers fall off from the side of the epitaxial and form cracks at the edge of the electrode, so the step becomes more obvious. The above results reveal that, for the double channel GaN/AlGaN epitaxial layer, the metal electrode of Ti/Al/Ni/Au system can obtain the optimal side ohmic contact characteristics at the annealing temperature of 750 °C. This kind of side ohmic contact mode is, hopefully, further used in the preparation of source and drain electrodes of double channel GaN HEMTs to improve their output characteristic.

## 5. Conclusions

A side ohmic contact mode for a double channel GaN/AlGaN epitaxial layer is proposed in this paper. The TLM electrodes with a side ohmic contact mode were prepared and annealed at temperatures from 700 °C to 850 °C. The results of specific contact resistance, alone with the analysis of SEM, EDS, and AMF, show that side ohmic contact mode for the double channel GaN/AlGaN epitaxial layer can obtain smaller specific contact resistance than the conventional surface contact mode at a lower annealing temperature. The calculation results of the specific contact resistance show that the minimum specific contact resistance of 2.58 × 10^−7^ Ω·cm^2^ is obtained at the annealing temperature of 750 °C. The observation of SEM, together with the EDS spectra, show that with the increase in annealing temperature, the state of the stress in the electrode films changes from compressive stress to tensile stress, leading to the volume expansion in the electrode films, and the composition of N will increase because of the creation of TiN and the N vacancies. When the annealing temperature is above 800 °C, cracks and lift-off of electrodes appear at the contact edge, which leads to a degradation of the ohmic contact performance. The AFM results reveal that, as the high temperature annealing causes the volume expansion of the metal layers, the step height decreases with the raise of the annealing temperature, while the RMS at the contact edge raises with the increase in annealing temperature, as the alloying of metal layers and the formation of grains at the contact edge begin. The experimental results prove that the proposed side ohmic mode has potential for application in double channel GaN/AlGaN HEMTs.

## Figures and Tables

**Table 1:** Main parameters of the ICP etching process.

Cl_2_ Flow (sccm)	BCl_3_ Flow (sccm)	Ar Flow (sccm)	RF Power (W)	ICP Power (W)	Etching Time (min)	Etching Depth (nm)
5	10	50	50	1500	5	120

## Data Availability

Not applicable.
